# Application of CIPP flipped lining method in the rehabilitation of old gas pipelines

**DOI:** 10.1038/s41598-025-95155-y

**Published:** 2025-04-01

**Authors:** You Yun, Wang xinxin, Wang Zhongyi, Wang Yuyan, Chen Hao

**Affiliations:** School of Petroleum Engineering, Chongqing University of Science and Technology, Chongqing, 401331 China

**Keywords:** CIPP flipped lining, Old gas pipelines, Trenchless, Repair techniques, Construction, Environmental sciences, Energy science and technology

## Abstract

With the increase of urban gas pipeline operation time, more and more underground gas pipelines are facing various problems, and old gas pipelines urgently need to be updated and repaired. Realizing the comprehensive excavation, demolition, and replacement of old pipes with new ones poses pain points such as high construction difficulty, repair costs, and uncertainty of routing construction planning. Therefore, the implementation of trenchless repair and transformation of old gas pipelines has positive and practical significance. The CIPP flipped lining repair technology, as an emerging trenchless pipeline repair technology, provides an efficient and cost-effective solution for the repair of old gas pipelines. A case study on the application of CIPP flipped lining method was conducted for the rehabilitation project of Liangshen Road gas pipeline in Weituo Industrial Park, Chongqing. By comparing the characteristics of different trenchless pipeline repair techniques, the technical adaptability of CIPP flipped lining method was analyzed, and the construction parameter design, key process flow, and construction difficulties of using CIPP flipped lining to repair gas pipelines were explored and revealed, as well as the control of engineering quality impact. Practice has shown that CIPP flipped lining can provide a feasible technical choice for the preventive repair of urban gas pipelines. The repaired pipeline performance meets the operational requirements of urban gas pipelines, and has broad prospects and promotion value. It can provide reference for the non-excavation repair and utilization of old gas pipelines in the future.

## Introduction

With the steady development of urbanization throughout the country, the scale of gas consumption is increasing day by day. Currently, the national gas-using population exceeds 667 million people, and the penetration rate of urban gas usage has reached 97.87%. Gas safety is related to thousands of households. In accordance with the requirements of documents such as the “Notice of the State Council’s Safety Production Committee on Printing and Distributing the ‘National Urban Gas Safety Special Rectification Work Plan’” (Safety Committee [2023] No. 3), the “Notice of the General Office of the State Council on Printing and Distributing the Implementation Plan for the Renovation and Upgrading of Aging Urban Gas Pipelines (2022–2025)” (General Office [2022] No. 22), and the “Notice of the Office of the Ministry of Housing and Urban-Rural Development and the Office of the National Development and Reform Commission on Further Clarifying the Requirements for the Renovation and Upgrading of Aging Urban Gas Pipelines” (Urban Construction Office [2022] No. 336), it is necessary to accelerate the renovation and upgrading of old gas pipelines, focusing on the governance of gas pipelines with safety hazards. Most of the old gas pipelines are buried under the existing roads in urban areas. The engineering workload of comprehensive excavation and removal of old pipes and replacement with new ones is enormous, especially the problems caused by open-cutting, such as roadbed damage, demolition and restoration of above-ground objects, environmental pollution, traffic congestion, etc., which lead to high construction difficulty, high repair costs, and uncertainty in routing construction planning. Adopting non-excavation in-situ pipe repair is an effective means to achieve rapid and efficient renovation of old gas pipelines. Among them, the CIPP (Cured-in-Place Pipe) flipping lining method involves inverting a prefabricated tubular composite lining material into the cleaned pipeline to be repaired and using adhesives to bond it tightly to the inner wall of the pipe, forming a high-strength lining new pipe. Compared with other repair methods, CIPP flipped lining repair avoids excavation operations during construction, and has obvious advantages such as strong adaptability, short construction period, energy saving and environmental protection, high durability, and good safety. This paper takes the renovation project of old gas pipelines on Liangshen Road in the Weituo Industrial Park in Chongqing as an example to explore the applicability, construction design, process, and quality control of using CIPP flipped lining to repair gas pipelines, providing a reference for the future trenchless repair and utilization of old gas pipelines.

## Project overview

**Fig. 1:**
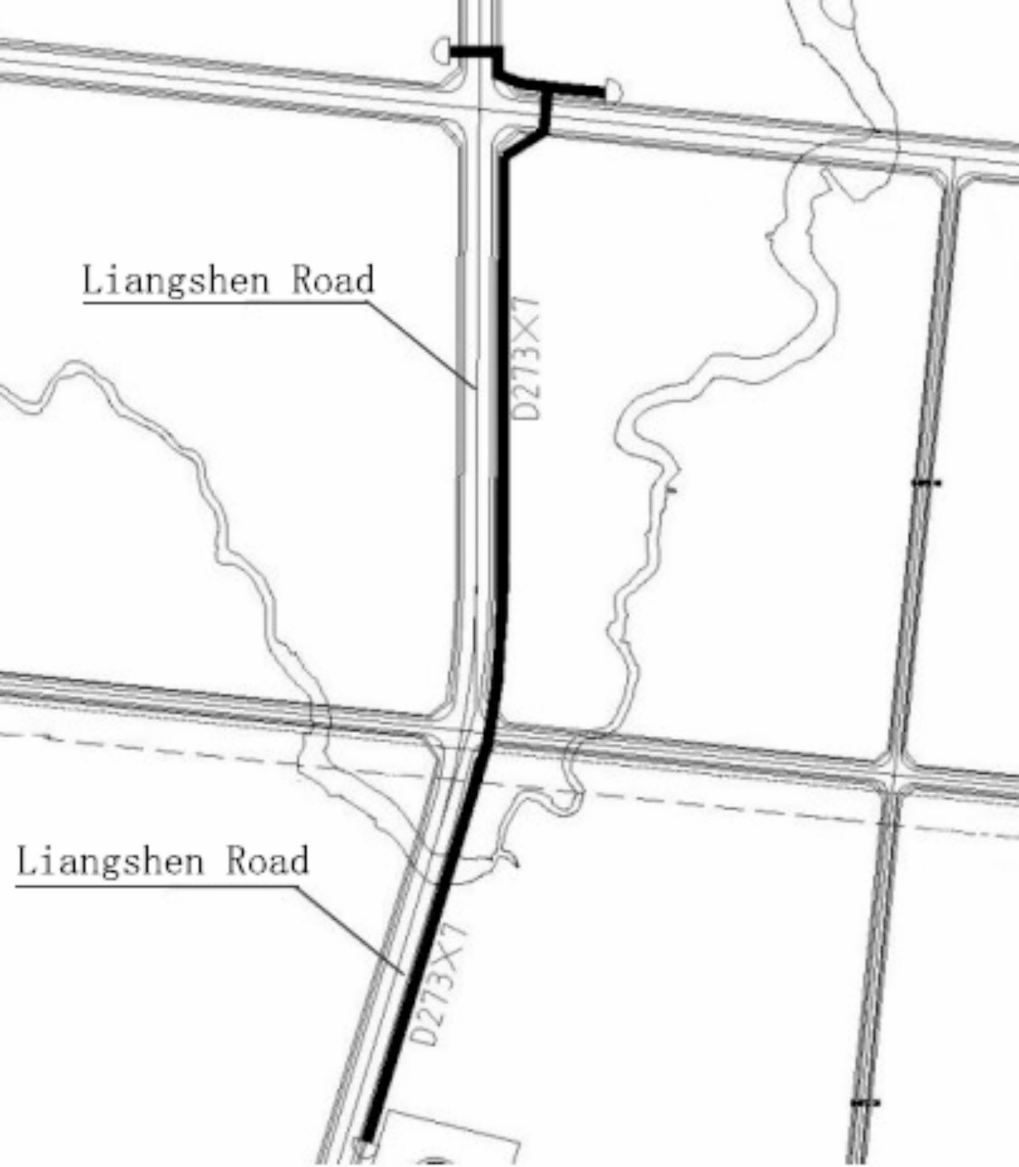
Gas pipeline routing diagram.

The gas pipeline project on Liangshen Road in the Weituo Industrial Park of Chongqing is designed with a total length of 1200 m, a horizontal length of 1148.25 m, and a design pressure of 0.8 MPa. The pipeline is intended to supply a gas scale of 40 × 10^4^ m^3^/d. It includes one valve well and utilizes seamless steel pipes with a specification of D273 × 7 mm. Due to topographical constraints, the pipeline features multiple bends or elbows at horizontal and longitudinal corners, as depicted in Fig. 1. At the time of construction, the road development and pipeline layout did not fully account for the industrial changes within the park. Consequently, the pipeline has undergone several route adjustments and depth modifications due to changes in the park’s planning. Moreover, effective routine maintenance and periodic inspections have not been conducted since the completion of the pipeline, leaving the internal smoothness and cleanliness of the pipeline uncertain.

Nowadays, the vicinity of the intersection at X617 and Tea Garden Avenue is characterized by a dense network interplay of various specialized underground pipelines, including gas, water supply, power, and communication conduits, leading to particularly scarce underground space resources. The traditional method of excavation for the repair and rectification of existing pipelines not only poses a threat to the surrounding environment but also encounters challenges such as difficulties in route selection, which are issues that are hard to accept for gas supply enterprises, management departments, and various societal sectors. To address these concerns, the project employs the non-excavation CIPP (Cured-In-Place Pipe) lining technology to rehabilitate and protect the existing pipelines, thereby extending the service life of the gas pipelines.The basic information investigation of the pipeline is shown in Table 1.

**Table 1:** Investigation of basic pipeline information.

Number	Information content	Survey results
One	Completion time	2013
Two	Original construction method	Excavation, trench burial and laying
Three	Pressure	0.8 MPa
Four	Pipe diameter	D273 × 7 mm
Five	Burial depth	1.2 m
Six	Tubing	20 # seamless steel pipe
Seven	Interface form	welding
Eight	Geological conditions	Good, no settlement or collapse, no standing water
Nine	Design traffic	40 × 10^4^ N m^3^/d
Ten	Current traffic	0 N m^3^/d
Eleven	Surrounding and road conditions	Park Road, 2-way 6-lane
Twelve	Maintenance situation	Unscheduled maintenance

## Applicability of CIPP flipped lining technology

The CIPP flipped lining rehabilitation technology uses a pipeline inspection robot to perform endoscopic inspection on old gas pipelines and clean the inner walls of the pipelines with sandblasting or high-pressure water jetting. Then, a glue applicator is used to evenly coat the interior of the lined hoses, which are then rolled up with a specialized flipping device. Finally, compressed air is used to flip the lined hoses onto the inner walls of the old pipelines, and after a period of curing, they are bonded together to form a new composite pipeline.

Compared to traditional excavation projects, the CIPP flipped lining method only requires the construction of a working pit to carry out the rehabilitation of old pipelines, thereby saving a series of processes such as road occupation approval, excavation, enclosure, traffic organization, backfilling, and maintenance, greatly improving construction efficiency and reducing the impact on the surrounding environment. Meanwhile, compared with other common trenchless pipeline rehabilitation techniques used for urban gas pipelines, as shown in Table 2.

**Table 2:** Comparison of different Non excavation repair techniques.

Number	Technology type	Flip lining method	Interlacing method	Folding lining method		Shrinkage lining method	Static pressure cracking method
				On site folding	Prefabricated folding		
One	Applicable pipeline diameter (mm)	200–600	80–600	100–400	100–500	100–500	100–400
Two	Inner lining tube material	Fiberglass, needle felt, resin, etc.	PE, PVC, fiberglass, metal pipes, etc.	MDPE/HDPE	MDPE/HDPE	MDPE/HDPE	MDPE/HDPE
Three	The relationship between the outer diameter d_N_ of the new pipeline and the inner diameter d_O_ of the old pipeline	d_N_ = 0.98d_O_	d_N_ ≤ 0.9d_O_	0.98d_O_ ≤ d_N_ ≤ 0.99d_O_	d_N_ ≤ 0.98d_O_	0.9d_O_ ≤ d_N_ ≤ 1.04d_O_	d_N_≤d_O_+100 mm
Four	Maximum suitable length for segmented construction (m)	five hundred	Five hundred	Three hundred	five hundred	three hundred	–
Five	Do you need a dedicated work pit	No need	Need	No need	No need	No need	No need
Six	Do we need grouting	No need	According to design requirements	No need	No need	No need	No need
Seven	Maximum allowable turning angle	45°	0°	45°	45°	15°	7°
Eight	Repair the original cross-sectional shape of the pipeline	Circles, rectangles, etc.	Rotundity	Rotundity	Rotundity	Rotundity	Rotundity
Nine	Efficiency	High	Lower	Higher	Higher	Higher	Higher
Ten	Price	Secondary	Secondary	Lower	Lower	Higher	Lower
Eleven	Applicable pressure	High, medium, and low pressure	Medium voltage	Medium voltage	Medium voltage	Medium and low pressure	Medium and low pressure

From this, it can be seen that the advantages and characteristics of using CIPP flipped lining method are:

1. Feasibility: Minimize excavation operations and demolition compensation in difficult areas such as traffic intersections, bustling neighborhoods, railways, and rivers, reduce costs, reduce the risk of damaging surrounding pipelines, and reduce overall engineering costs by 20%.
2. Technicality: Can repair high temperature and high pressure pipelines; Can continuously bend; Can prevent internal corrosion; After repair, the traffic can be increased by 30%; Can extend pipeline lifespan by 30 years.
3. Timeline: Avoid excavation in locations with structures or obstacles on the ground or underground, and the maximum construction period can be shortened by 60%.
4. Cost: The compensation costs for excavation and demolition are reduced, the risk of damaging surrounding pipelines is reduced, and the overall cost of the project can be reduced by 20%.
5. Environmental friendliness: Reduce carbon emissions by more than 90%; The construction process generates less dust, operates with lower noise levels, and minimizes the disruption to green spaces.

## Wall thickness design and material prefabrication of inner lining pipes

### Inner lining pipe wall thickness

At present, there are no national standards or industry technical specifications specifically for CIPP flipped lining in urban gas pipelines, and with only some local standards available in places like Beijing. In this regard, based on relevant foreign technical standards and experience, in order to meet the requirements of compressive and flow capacity after gas pipeline repair, the circular pipe bending formula is adopted for the design of the inner lining pipe structure. By referring to the relevant calculation methods in ASTM 1216, ASTM 1743, and ASTM 2019 of the American Society for Testing and Materials, the minimum wall thickness of the inner lining pipe is calculated as follows:

1 $$t = \frac{{D_{0} }}{{\left[ {\frac{{2KE_{L} C}}{{\left( {P_{w} + P_{v} } \right)N\left( {1 - \mu ^{2} } \right)}}} \right]^{{\frac{1}{3}}} + 1}}$$

2 $$C={\left[ {\frac{{\left( {1 - \frac{q}{{100}}} \right)}}{{{{\left( {1+\frac{q}{{100}}} \right)}^2}}}} \right]^3}$$

3 $$q = 100 \times \frac{{D_{{max}} - D_{E} }}{{D_{E} }}\;\;q = 100 \times \frac{{D_{E} - D_{{min}} }}{{D_{E} }}$$

In the formula, t—the calculated thickness of the pipe wall (mm); Pw—groundwater pressure at the bottom of the pipe (MPa), Pw = 0.00981 H; H—depth of groundwater level at the bottom of the pipe (m); Pv—vacuum pressure (MPa) (based on actual engineering values and not less than 0.05 MPa); N—resistance coefficient for circumferential stability of pipeline cross-section (value should not be less than 2.0); ${{\text{E}}_{\text{L}}}$—the long-term bending elastic modulus (MPa) of the inner lining pipe should be selected according to 50% of the short-term bending elastic modulus in Table 3, and the inner lining material should be 1968 MPa; K—circle support rate, can be taken as 7.0; µ—poisson’s ratio, can be taken as 0.3; C—ellipticity reduction factor; q—ovality of the original pipeline (%); ${{\text{D}}_0}$—calculated diameter of pipeline (mm); ${{\text{D}}_{\text{E}}}$—the average inner diameter of the original pipeline (mm); ${{\text{D}}_{{\text{min}}}}$—the minimum inner diameter of the original pipeline (mm); ${{\text{D}}_{{\text{max}}}}$—the maximum inner diameter of the original pipeline (mm).

**Table 3:** Initial mechanical performance indicators of inner lining tubes.

Performance	Index	
	Ordinary felt inner lining tube	Fiberglass inner lining tube
Bending strength (MPa)	≥ 31	≥ 45
Short term bending modulus of elasticity (MPa)	≥ 1724	≥ 6500
Tensile strength (MPa)	≥ 21	≥ 62

Regarding this, the values and calculations of the design parameters for the inner lining pipe wall thickness in this project are shown in Table 4.

**Table 4:** Values and calculation results of inner lining tube thickness parameters.

Parameter	${D_0}$ /mm	K	${E_L}$ /MPa	C	Pv /MPa	*N*	$\mu$	*q*	${D_E}$	${D_{max}}$	D_min_	*P*_w_	t /mm
Value/calculated value	273	7	1968	1	0.06	2	0.3	0	273	273	273	0.03	4.86

After calculation, the wall thickness of the inner lining pipe is 4.86 mm, which is rounded to a value of 5 mm.

### Preparation of inner lining tubes

At this stage, the lining hose materials used for trenchless repair are mostly high-performance composite materials. The lined hose is generally divided into a base layer and an anti-seepage layer. Among them, the anti-seepage layer is a fabric membrane structure, one fabric is non-woven fabric, and the other membrane is an anti-seepage membrane. The anti-seepage film in the composition of the hose can ensure that the resin material is sealed in the hose skeleton without leakage, and can also improve the surface performance of the repaired pipe wall, making the repaired pipe wall smooth and improving the flowability of the pipeline^1^. The anti-seepage membrane mainly uses linear low-density polyethylene (LLDPE) or thermoplastic polyurethane (TPU), both of which are thermoplastic materials.

**Fig. 2:**
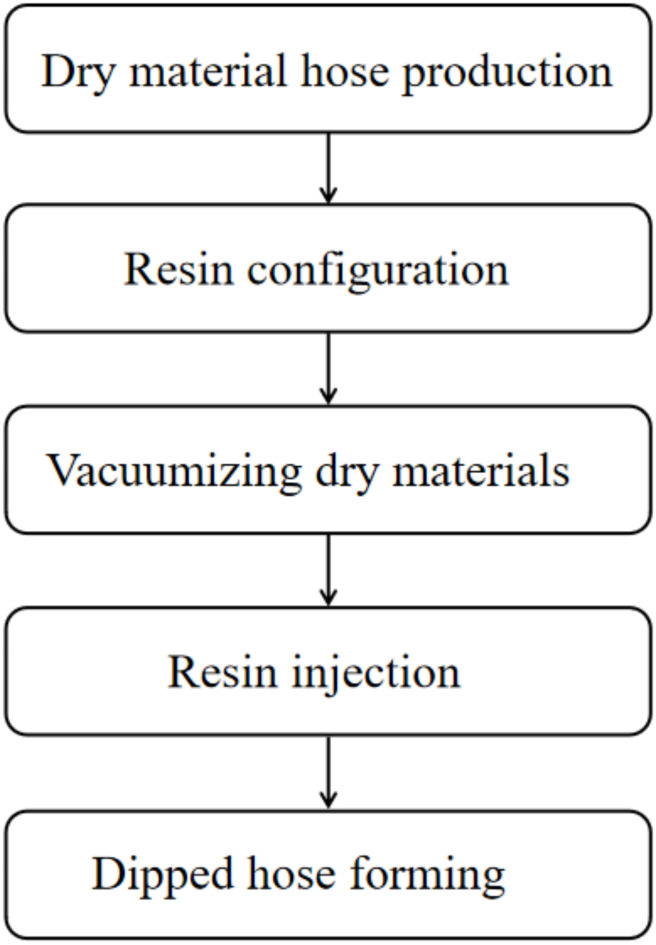
Production process of lined hose.

The production process of the inner lining pipe is shown in Fig. 2. In the prefabrication factory, the protective film, outer film, fiber cloth, and inner film are gradually folded through equipment, and the outer film and protective film are fused to produce a tubular composite lining material. The end of the dry material hose made is vacuumed using a suction cup, which extracts the air inside the dry material hose to form negative pressure, so that the resin and curing agent can be evenly mixed in a reasonable proportion and injected^2^. When resin is poured, it starts from the first section of the dry material. Under vacuum action, the resin wets from one section of the dry material to the other end. To accelerate the resin infiltration speed, the resin can be fully and evenly impregnated into the fiber cloth through roller squeezing^3^.

Hoses treated with resin impregnation are stored in an environment with a temperature below 20℃, avoiding direct sunlight and high temperatures, ensuring that quality and performance are not affected^4^. In the subsequent transportation process, the hose must also be kept refrigerated throughout the entire process and sealed to prevent mechanical damage, chemical corrosion, and external factors from damaging it, to ensure good performance before use.

## CIPP lining construction process and technical requirements

### Construction process flow

**Fig. 3:**
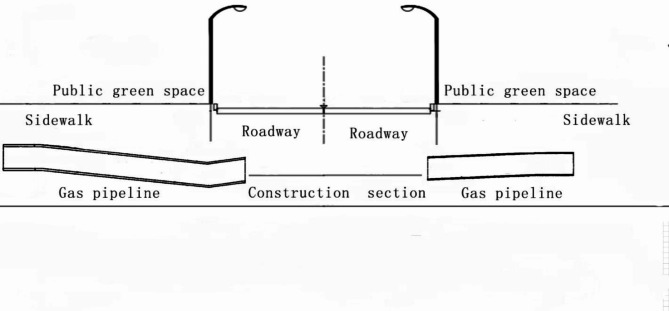
Schematic diagram of the CIPP inversion lining construction section.

The CIPP lining inversion method for repairing gas pipelines is carried out under the construction conditions as shown in Fig. 3.

**Fig. 4:**
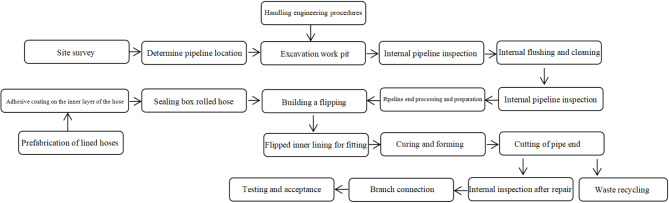
Construction process flow.

The construction process of repairing gas pipelines with CIPP flipped inner lining is shown in Fig. 4. The project excavates small work pits on both sides of the pedestrian walkway at the intersection of Yaoyuan Avenue and Shugang Avenue, cuts open the original gas pipeline, and uses a pipeline inspection robot to perform pipeline inspection. Then, a pipeline cleaning system is used to clean the impurities inside the gas pipeline. The high-pressure gas provided by a high-power air compressor is used to flip the inner lining hose, and after flipping is completed, the pressure is maintained for a period of time to ensure adhesive curing, allowing the lining soft pipe to fully adhere to the gas pipeline, and finally use special cutting tools to remove excess solidified inner lining hose from the pipeline end. The pipeline is welded, the working pits are backfilled, and the work is inspected and tested for quality assurance.

### Key construction links

#### Excavation of work pits

The main focus of the project construction layout work is on setting up work pits. The location of the work pit takes into account the safety of surrounding buildings and traffic impacts caused by excavation, as well as the special requirements of non-excavation repair and renewal projects for the work pit. The project fully utilizes the terrain and combines with the transportation routes inside and outside the site, making reasonable use of effective space, and chooses to set up work pits at the shoulders on both sides of the cross road, that is, excavate one at each end of the gas pipeline crossing section, and excavate a guide groove with a slope of about 20° on one side of the operation pit. According to the on-site conditions of the gas pipeline, the work pit is excavated and the bottom surface is treated. The broken length of the original gas pipeline is about 2.5 m, and the exposed section of the pipeline is not less than 0.5 m. The ends are neat and smooth, and burrs are polished off.

#### Flip operation

**Fig. 5:**
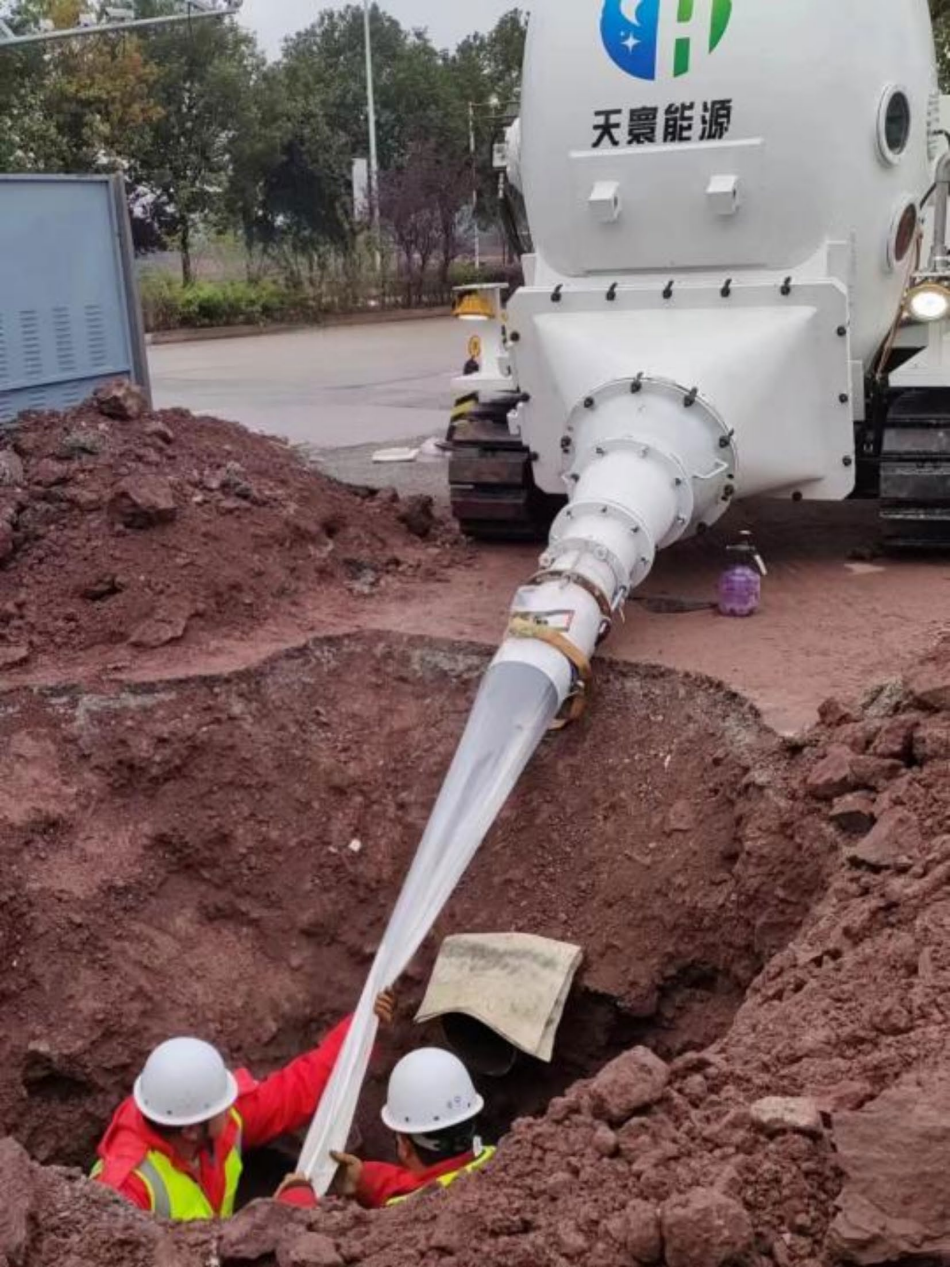
Flip carriage entry into operation.

Pre-roll the inner lining hose coated with adhesive into the compartment of the inner lining pipe flipping chamber, and then drive it to the working pit at the construction site. Start the air compressor unit to flip the inner lining pipe in the chamber into the old and cleaned pipes that have passed the inspection, as shown in Fig. 5. Control the temperature of the flipping chamber to be less than 20 °C to prevent premature curing reaction of the resin due to excessive temperature. The entire process of flipping the lining relies on the action of compressed fluid to push the flipping head forward in the original pipeline. During the process, attention should be paid to real-time monitoring of flipping pressure and control of flipping speed. The flipping pressure is controlled within 0.05 –0.1 MPa to ensure that the hose can fully expand during the flipping process without exceeding its bearing capacity limit. The flipping speed should be controlled at 2 m/min to 3 m/min, and special attention should be paid to the pushing situation when the inner lining pipe passes through the bends or elbows during the feeding process. The flipping speed of the inner lining hose is approximately linearly related to air pressure. Maintaining a constant speed of the flipping head is a key step in ensuring that the inner lining material can be uniformly adhered and avoiding wrinkles and bubbles^5^. If the speed is too fast, problems such as folding, wrinkling, and insufficient space for tight fitting of the inner lining hose may occur. On the other hand, if the speed is too slow, it will cause the adhesive to flow to the bottom of the pipe due to excessive stock, resulting in inconsistent thickness of the inner lining inside the pipe, thereby affecting the repair effect and the overall performance of the pipeline. After flipping, the length of the resin impregnated hose extending from both ends of the original pipeline should be greater than 1 m^6^.

#### Curing and forming

After confirming that the inner lining pipe is flipped to the other end of the gas pipeline, install the end flange blind plate, increase the pressure of the entire line to 0.08 MPa, and close the knife valve and air compressor. Sufficient curing time is crucial for ensuring the quality of the repair, to ensure that the inner lining hose can fully stretch and tightly adhere to the original pipeline inner wall during the curing period, preventing gaps that may lead to inner wall wrinkles. Usually, the lower the ambient temperature, the longer the curing time is required. To artificially shorten the curing time, catalysts can be added to shorten it. The soil temperature during the construction of this project is 20℃, and the solidification time will last for 72 h according to Table 5. During the entire curing period, the pressure inside the pipe remains positive, causing the inner lining pipe to come into close contact with the original pipeline. Due to the fact that the curing process itself is a violent exothermic reaction^7^. This requires attention to the temperature changes inside the pipe during construction, to avoid heat accumulation on the inner wall of the lined hose in some positions, which may cause damage to the prefabricated structure of the lined hose and affect the surface morphology interface of the cured “new” pipe inner wall. After confirming the curing process, wait for the temperature inside the tube to naturally cool down and slowly release pressure to prevent shrinkage cracks in the inner lining tube^8^.

**Table 5:** Curing schedule.

Room temperature curing		Accelerated curing	
Soil temperature °C	Minimum curing time h	Soil temperature °C	Minimum curing time h
25	48	≥ 10	24
20	72	< 10	24
15	96	Additional catalyst needs to be added	
10	120		

#### End treatment

**Fig. 6:**
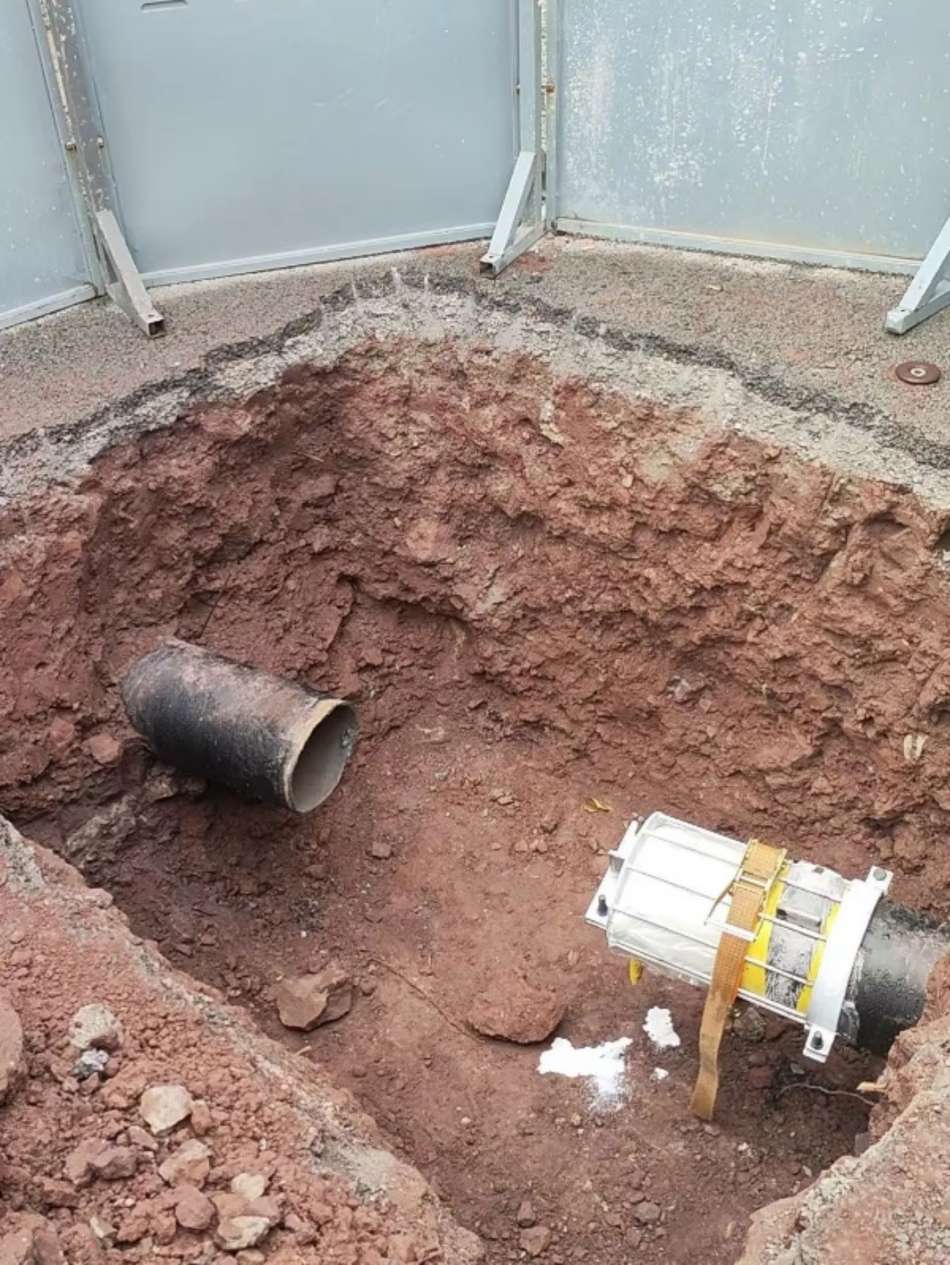
Pipeline end.

The end treatment mainly includes sealing and cutting work. After cutting, the burrs at both ends are trimmed and treated, and applying a quick-setting waterproof material at the interface between the inner lining pipe and the inspection well to fully ensure that there is no leakage between the inner lining pipe and the old pipeline. Remove the devices at both ends and use special tools to strip off the inner lining of about 20 cm on both ends, making sure the cuts are neat. When the inner lining pipe is not tightly attached to the original pipeline, a resin mixture should be filled for sealing^9^. Then weld the short pipes of the inner lining work pit in sequence, and protect the completed inner lining section while welding to avoid damaging the inner lining material at the welding points. The welding standard shall comply with the Construction and Acceptance Specification for Industrial Pipeline Welding Engineering of Field Equipment (GB50236). As shown in Fig. 6.

## Quality assessment of repaired gas pipelines

The CIPP flipped lining method lacks a unified monitoring method and standard for the quality of repaired gas pipelines in the absence of current industry technical specifications. The project refers to the industry standard “Urban Gas Transmission and Distribution Engineering Construction and Acceptance Standards” (GB/T51455-2023) for strength and tightness tests on the repaired pipelines. Additionally, it draws on standards from the American Society for Testing and Materials (ASTM) and “Technical Specifications for Non-Excavation Repair and Renewal of Urban Gas Pipelines” (CJJ/T 147) for the quality inspection, evaluation, and acceptance of the lining pipe, mainly including:

1. Certificate of conformity, quality certification documents, and inspection reports from third-party inspection agencies for lining materials.
2. The thickness of the inner lining tube should meet the design requirements, and the design thickness error of the inner lining tube should be within the allowable range.
3. CCTV pipeline endoscopic inspection video data for pipeline cleaning and flipping inner lining construction process, ensuring that the inner wall of the flipped repaired pipeline should be smooth and tidy, without solidification, bulging and layering, and without cracks and serious wrinkles.
4. After repair, a test block was taken from the end of the inner lining of the pipeline for third-party testing, as shown in Table 6.

**Table 6:** Third party testing results of lining materials.

Number	Project			Unit	Technical indicators	Detection result
One	Mass per unit area			mg/cm^2^	/	181.33
Two	Breaking strength		Radial	N/cm	≥ 800	959
Three			Latitudinal direction	%	≥ 800	1113
Four	Elongation at break		Radial	%	≥ 20	21
Five			Latitudinal direction	%	≥ 20	28
Six	Aging performance (30 d, 70 °C)	Change rate of fracture strength	Radial	%	– 25 to 50	29
Seven			Latitudinal direction	%	– 25 to 50	16.1
Eight		Elongation at break	Radial	%	– 25 to 50	9.5
Nine			Latitudinal direction	%	– 25 to 50	32
Ten	Gas resistant components (28 d, 23 °C)	Change rate of fracture strength	Radial	%	< 20	1.8
Eleven			Latitudinal direction	%	< 20	11.3
Twelve		Elongation at break	Radial	%	< 20	4.8
Thirteen			Latitudinal direction	%	< 20	19.5
Fourteen		Mass retention rate per unit area after soaking and drying		%	≥ 95	97.7

## Conclusion

The CIPP flipped lining repair technology, as an emerging non-excavation pipeline rehabilitation technology, provides an efficient and economical solution for the repair of old gas pipelines, with broad prospects and promotion value. The practical application of CIPP flipped lining repair for gas pipelines in China is still in its early stages, and the relevant standards and regulations are not yet perfect. This article takes the repair project of the Liangshen Road gas pipeline crossing section in Chongqing Weituo Industrial Park as the object, summarizes some of the experience gained during construction, and reveals the design of CIPP flipped lining construction parameters, process flow, and quality impact control of key links, in order to provide reference for similar projects.

1. The gas pipeline specification is D273 × 7 mm. After completion, there have been multiple changes in the route, resulting in a large number of horizontal and vertical bends or bends in the route. The situation of the route crossing the highway is more complex. After comparing various non-excavation repair techniques, the CIPP flipped inner lining repair process was adopted and successfully applied in the repair project of the Liangshen Road gas pipeline highway crossing section in Weituo Industrial Park, Chongqing.
2. The construction parameters and inner lining pipe wall thickness index of the CIPP repair project for gas pipelines have been clarified. After calculation and selection, the inner lining pipe thickness is designed to be 5 mm. The preferred tubular composite material is polyurethane film and textile fiber material, and the adhesive is epoxy resin, making the inner lining pipe more suitable for practical working conditions.
3. Based on the practical aspects of on-site implementation and management in engineering construction, this paper focuses on analyzing the construction difficulties of CIPP flipped lining repair technology, indicating that the level of construction operation technology in key processes greatly affects the quality of repair.
4. Through experimental testing, it has been shown that the inner wall surface of the lined hose has no bulging, cracks, or wrinkles, and the inner wall of the lined hose is tightly bonded to the original pipeline. Its performance meets the operational requirements of urban gas pipelines and meets the acceptance standards. This has played a demonstration role in promoting the flipped inner lining repair technology of gas pipelines^10^.

## Data Availability

The datasets used and/or analysed during the current study available from the corresponding author on reasonable request.
